# Genetic Diversity of *Orobanche crenata* Populations in Ethiopia Using Microsatellite Markers

**DOI:** 10.1155/2020/3202037

**Published:** 2020-08-13

**Authors:** Gashaw Belay, Kassahun Tesfaye, Aladdin Hamwieh, Seid Ahmed, Tiegist Dejene, José Oscar Lustosa de Oliveira Júnior

**Affiliations:** ^1^Amhara Agricultural Research Institute, Bahir Dar, Ethiopia; ^2^Institute of Biotechnology, Addis Ababa University Addis Ababa, Ethiopia; ^3^Ethiopian Biotechnology Institute (EBTi), Addis Ababa, Ethiopia; ^4^International Center of Agricultural Research in the Dry Areas, Rabat, Morocco; ^5^College of Agriculture and Environmental Sciences, Bahir Dar University, Bahir Dar, Ethiopia; ^6^EMBRAPA Mid-North, Brazilian Agricultural Research Corporation (EMBRAPA), Teresina PI-, Brazil

## Abstract

*Orobanche crenata* is a parasitic weed that causes considerable yield losses on food legumes in Ethiopia and the Mediterranean region. Understanding the genetic diversity of *Orobanche crenata* using molecular techniques generate useful information in managing the weed through resistance breeding. This study aimed at assessing the genetic diversity of *O. crenata* populations collected from major faba bean growing areas of Ethiopia. A total of 96 samples were collected from the Orobanche-infested faba bean farmer field. The genetic diversity of the population was studied using 30 *O. cumana* SSR markers. The results showed that 11 SSRs were functional and transferable markers to study the diversity of *O. crenata* populations. The average number of alleles, gene diversity, heterozygosity, and polymorphic information content values for the SSR loci were 9.6, 0.82, 0.38, and 0.80, respectively. The pairwise genetic similarity analysis showed the lowest genetic distance between samples collected from South Gondar and South Wollo (0.12) while the highest genetic distance (0.48) was found between South Gondar and North Wollo. The analysis of molecular variance result indicated that the variation among individuals was a major source of genetic variation (55%) followed by within individuals (43%) and among populations (2%) variation. The output of population genetic structure analysis indicated the presence of two major groups irrespective of the area of collection or region of origin. Besides, the outcome of the spatial autocorrelation computation indicated a significant and positive genetic correlation between samples collected under a 28 km radius. In general, the absence of geographic region based genetic structure presumably demonstrates the expansion of the parasitic weed between farming sites upon its recent introduction to the country. Thus, the clear absence of population differentiation warrants screening faba bean population in hot spot area.

## 1. Introduction

The parasitic weed (*Orobanche* spp.) imposes considerable yield losses on faba bean (*Vicia faba*), lentil (*Lens culinaris*), carrot (*Daucus carota*), pea (*Pisum sativum*), chickpea (*Cicer arietinum*), and vetches (*Vicia* spp.) [1]. Broomrapes (*Orobanche spp.*) are native to the Mediterranean region (North Africa, the Middle East, and southern Europe) and western Asia [2]. *Orobanche crenata* is an obligate root parasite, which is widely distributed in the Mediterranean region, the Middle East, and Eastern Europe [3]. *O. crenata* has diploid chromosome numbers (*n* = 19, 2*n* = 38) [4, 5].

Faba bean (*Vicia faba* L.) is an important cool-season food legume in the highlands of Ethiopia. The crop is used for food, animal feed, income generations, and improving soil fertility. Despite its importance, the productivity of faba bean is below 2 t ha^−1^, which is caused due to low yielding landraces, parasitic weeds (*Orobanche crenata* Forsk), and diseases [6–8]. *Orobanche crenata* was first reported in 1993 in one village on 10 ha of faba bean crop in the north East Amhara Region of Ethiopia [9]. Previous report indicates that O. crenata is suspected to occur in Ethiopia with alarming expansion where food legumes are the major production area [10]. Currently, the weed covers many districts in Amhara and Tigray regions causing up to 100% yield losses ([8, 11]). Besides crop and straw losses, the weed is believed to be causing genetic erosion since the local landraces are highly susceptible.

Not much attention have given by researchers and extension staff to manage parasitic weeds for a long period of time, and many farmers have abandoned growing cool-season food legumes, and especially faba bean. Recently, one partially resistant faba bean cultivar Ashengie (ILB-4358) was released from ICARDA germplasm sources. The genetic diversity of *O. crenata* population affecting faba bean is not known in Ethiopia except few surveys to determine its distribution and yield loss [10].

Genetic diversity of *O. crenata* populations were studied using RAPD markers showed low genetic variation among populations in southern Spain [12, 13], while ISSR markers detected more diversity with clear differentiation among populations.

The patterns of genetic variation among *Orobanche crenata* populations from Spain and Israel have been studied using five ISSR (Inter Simple Sequence Repeat) and one RAPD (OPG-06) markers, as a result, significant divergences were found between regions with more differences among individuals within a population [12]. Moreover, a dendrogram divided the six populations by region, with the Spanish populations being more similar than the Israeli populations [12].

Understanding the genetic diversity and population structure *O. crenata* within and among populations is an important source of information for the national breeding activity to develop faba bean cultivars resistant to the pest as well as design appropriate control measures. In Ethiopia, there is a knowledge gap on the status and pattern of genetic diversity of *O. crenata* populations in various legume growing areas of the country. To our knowledge, this study is the first to look at the genetic diversity of *O. crenata* using molecular markers in Ethiopia and, therefore, will be an important starting point for further studies of this parasitic weed. Hence, the present study was carried out to test the transferability of SSR markers from *O. cumana* and assess the genetic diversity of *O. crenata* population in Ethiopia.

## 2. Materials and Methods

### 2.1. Sampling and Plant Materials

**Figure 1:**
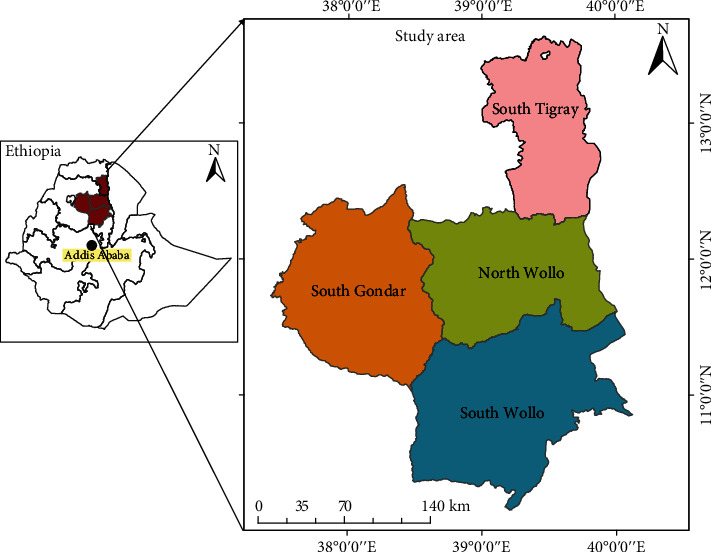
Map showing the study areas on parasitic weeds affecting food legumes in North Ethiopia.

A multistage purposive sampling technique was followed to randomly sample shoot tip of *O .crenata* from infected faba bean, field pea, and other legumes field. A total of 96 *O. crenata* samples were collected from South Tigray (30), South Wollo (31), North Wollo (5), and South Gondar (30) (Figure 1). Individual samples of *O. crenata* (2-3 cm long shoot tips) were collected, sliced with a blade, and then dried using silica gel for DNA extraction.

### 2.2. Genomic DNA Extraction

Genomic DNA of *O. crenata* was extracted from silica gel dried shoot tips using a modified CTAB method [14]. Approximately equal amount (0.15 g) of dried single shoot tip samples was grounded with Mix and Mill grinding machine MM 400 and ready for downstream DNA extraction process following Borsch et al. [14]. DNA isolation was performed at Plant Genetics Research Laboratory, Addis Ababa University, Ethiopia. The extracted DNA was tested on agarose gel (0.8% *w*/*v*) and visualized using the Bio-Rad gel doc system, after running 45 min at 100 V and staining using RedSafe™ (Scientific, NSW, AUS).

The concentration and purity of extracted genomic DNA were quantified using NanoDrop (NanoDrop™2000/2000c) spectrophotometer, and the absorbance ratio of OD (260/280) was within a range of 1.8 to 2.1. The DNA samples were then adjusted to a concentration of 50 ng *μ*L^−1^ by diluting with double distilled sterilized water for SSR-PCR analysis.

### 2.3. SSR Marker Screening and Transferability

Thirty SSR markers with polymorphic information content (PIC) values ranging from 0.37-0.80 were selected from published SSR makers for *O. cumana* [15]. Marker transferability and genetic diversity analyses were done at the Biotechnology Laboratory of the International Center for Agricultural Research in the Dry Areas (ICARDA) at the Agricultural Genetic Engineering Research Institute (AGERI), Cairo, Egypt.

The SSR-PCR amplifications were carried out in 10 *μ*L reaction volumes containing 1 *μ*L template DNA, 1x buffer, 0.075 units *Taq* DNA polymerase, 0.25 mM dNTPs, and 0.5 pM each primer. Amplification was performed by using touchdown PCR program on Applied Biosystems: Veriti96 well Thermal Cycler, which consisted of an initial denaturation of 94°C for 2 min, followed by 1 cycle of 94°C for 30 s, final annealing temperature (Tm) +10°C for 30 s, and 72°C for 30 s, nine cycles in which the annealing temperature was decreased 1°C, and 32 cycles at 94°C for 30 s, Tm for 30 s, and 72°C for 30 s, with a final extension of 20 min at 72°C and then stored at 4°C.

Amplified PCR products were separated on 2% agarose gels in 1x TAE buffer with RedSafe Nucleic Acid Stain incorporated in the gel, and 50 bp DNA Ladder was used as a standard molecular weight marker to compare amplicons with their expected size. Microsatellite alleles were detected for their amplification and correctness; hence, true amplicons were subjected for QIAxcel advanced capillary system electrophoresis analysis (Qiagen) by using QIAxcel DNA High-Resolution Gel Cartridge (Cat No./ID: 929002).

### 2.4. Allele Scoring and Data Analysis

Fragment analysis from the raw data generated on QIAxcel capillary gel electrophoresis output was analyzed by using QIAxcel Screen Gel Software to determine and score allele peaks. Allelic data were used to compute polymorphic information content, observed heterozygosity, gene diversity, number, and frequency of alleles using PowerMarker V: 3.25 software [16].

To see variation among and within the studied populations, analysis of molecular variance (AMOVA) was computed using GenAlEx V6 [17]. Population genetic parameters such as the percentage of polymorphism, population differentiation were examined using Arlequin software [18].

Principal component analysis was carried out based on principal component values generated from 106 alleles of 11 SSR markers. The values were calculated using SAS ver. 9.3, using Princomp procedure. The first two principal components were used for the visualization of the samples on 2D scatter plot.

To understand the genetic structure and infer the possible number of subgroups, STRUCTURE software was used [19]. The analysis was computed using 50 000 iterations and 50 000 MCMC burn in for 10 independent runs from *K* = 1 to *K* = 10. The Evanno *et al.* [19] method was applied to determine the number of *K*. For this function, a web-based program, STRUCTURE HARVESTER ver. 0.9.93 [20] was employed to extract the optimum number of subgroups. Later, CLUMPP software [21] was used to combine individual membership coefficient generated from independent 10 runs, and later it was plotted using DISTRUCT software [22]. To identify samples that were admixed, each individual sample was assigned to its respective group based on a membership coefficient (*Q*). The threshold for the membership coefficient was 90%, in which samples sharing more than that were assigned to their respective subgroups, whereas those that were smaller than the threshold were considered admixed. Hence, the assignment of each sample to one group based on the membership coefficient used as criteria to group individuals on a scatter plot using the first two principal component values.

The spatial autocorrelation analysis was computed based on the similarity matrix calculated from 106 alleles of 11 SSR markers and geographic distance matrix generated from the coordinates of the samples. The analysis was conducted using the “Spatial” option implemented in GenAlEx ver. 6.41 [17]. The significance of the spatial autocorrelation value was tested by constructing a two-tailed 95% confidence interval around the null hypothesis of no spatial genetic structure, which is *r* = 0. The analysis was performed with an option of an even distance class of 10 km, and permutations of 9999 and a bootstrap of 1000 were used to compute the confidence interval around the null hypothesis.

## 3. Results

### 3.1. SSR Marker Transferability and Genetic Diversity

From the total of 30 SSR, markers evaluated and screened initially 11 SSR markers with fragment size ranged from 90-274 bp and PIC value ranged from 0.59 to 0.92 were selected (Table 1). These SSR markers resulted in a total of 106 alleles that ranged four (Ocum-063) to seventeen (Ocum-059) alleles with average heterozygosity of 0.38 per all loci.

The highest polymorphism percentage was observed for samples collected from South Wollo (72.73%) whereas samples from South Tigray scored the lowest percentage (45.45%) value. Accordingly, the highest numbers of alleles were counted for samples collected from South Wollo (8.82) and the least for samples from South Tigray (3.45). For all four regions, the mean allele frequency was scored as 7.25. The average genetic diversity for all sites was high (0.77), which ranged from 0.82 to 0.74 for South Wollo and North Wollo, respectively. The average observed heterozygosity (Ho) in the total sample was 0.37, which is significantly deviated from the expected heterozygosity (He) 0.78 (Table 2).

### 3.2. Analysis of Molecular Variance and Population Differentiation

The Analysis of Molecular Variation (AMOVA) showed that 2% of the variation resulted from the difference among populations, 55% of variation from among individuals, and 43% of the variation within individuals (Table 3). *F*-statistics indicated that low among population differentiation (0.026), differing from relatively high values for among individual (0.562), and within individual (0.573) differentiation (Table 4).

The pairwise *F*_ST_ among regions showed little (0.0-0.05) to moderate (0.05-0.15) genetic differentiation indicating high gene flow among population resulting in the absence of region-based population structuring. Among the collection areas, South Wollo showed little differentiation when paired with all collections sites except North Wollo (0.058). Another two pairs of regions: South Gondar and North Wollo (0.096); and South and North Wollo (0.092) showed moderate genetic differentiation (Table 5).

In the table, *F*_ST_ values are presented below diagonal. While probability, P(rand ≥ data) based on 999 permutations is shown above diagonal. This result indicates the presence of significant genetic differentiation between regions, despite little *F*_ST_ values.

### 3.3. Principal Component Analysis

**Figure 2:**
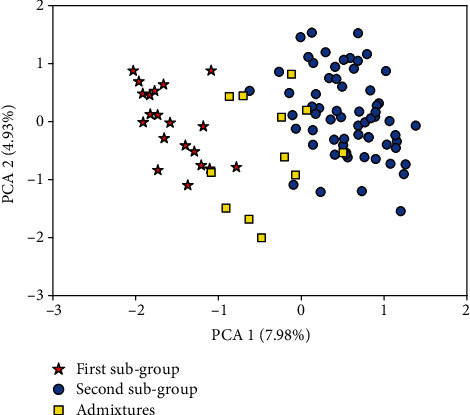
Principal Component analysis of Ethiopian *O. crenata* samples from different regions. Each type of symbol in the figure represents the subgroup; the sample was assigned based on the structure grouping result.

The figure presented below (Figure 2) is based on principal component values generated from 106 alleles of 11 SSR markers. The eigenvalues were used to plot the samples while the membership coefficient (*Q*) from the population structure was used to assign the individuals to their respective groups indicated in different shapes. The threshold for the membership coefficient was 90%, in which genotypes sharing more than that were assigned to their respective sub-groups. However, those individuals who failed to have this membership coefficient were considered admixtures and did not belong to any subgroups. The first two principal components explained 12.91% of the total variation (Figure 2). These two components were able to clearly distinguish the two groups (subgroup one and subgroup two) while the admixed individuals were scattered over the map. In general, the samples were not grouped based on their geographical based population.

### 3.4. Population Structure Analysis

**Figure 3:**
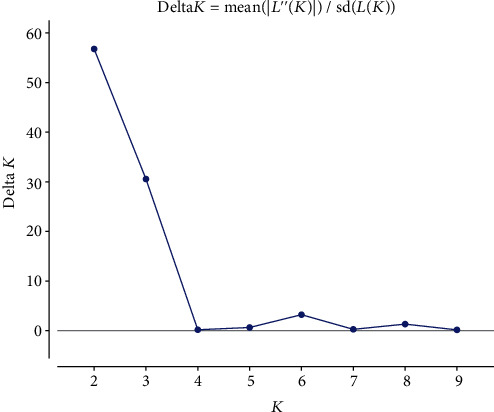
Structure estimation of the number of subgroups for *K* ranging from 1 to 10.

**Figure 4:**
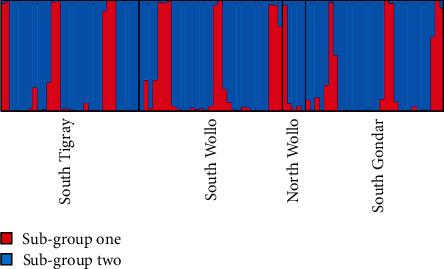
This figure shows the genetic assignment of individuals to populations inferred from structure analysis at *K* = 2 based on SSR markers.

The analysis of the population structure of the studied populations showed *K* = 2 as the optimum number of subgroups (Figure 3). Based on the membership coefficient (greater than 90%) assignment, 20.8% of 96 samples were assigned solely to the first subgroups. However, the majority of (66.7%) the samples were assigned purely to subgroup two. The rest of the samples (12. 5%) were considered admixed because of the commonly shared ancestors with individuals assigned to the detected subgroups. The composition of each individual was represented by two different colors, dark grey color represents subgroup one and light grey subgroup two (Figure 4). This result showed that both subgroups represented are by each individual, which have different membership coefficients, collected from all regions indicating the absence of geographic region based genetic structuring.

### 3.5. Spatial Autocorrelation Analysis

**Figure 5:**
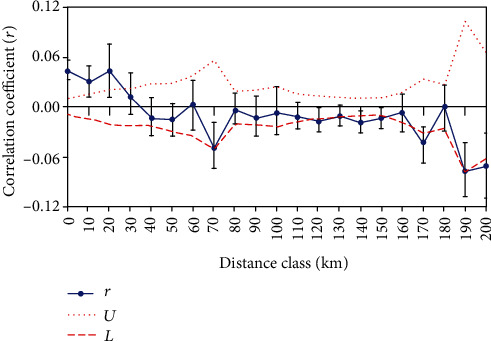
This graph indicates the correlation between the genetic similarity and the geographic distance matrices for Ethiopian O. crenata samples.

The spatial autocorrelation analysis result showed a significant correlation between genetic similarity and geographic distance for Orobanche samples collected in a range of 28 km. The Orobanche which was collected in an area of more than 28 km shows no correlation. Hence, as geographic distance increases beyond 28 km, the genetic similarity started to reduce. The broken lines indicate the upper and lower 95% confidence limits for the null hypothesis, which states is no correlation between the two matrices, and the solid line in the middle indicates the correlation coefficient (Mantel r) (Figure 5). The black lines are whiskers indicating the magnitude of variation. When the sampling distance started to increase, the mantel correlation coefficient became decreasing resulting in nonsignificant correlation. This result implies that when the geographic distance increases, the genetic similarity started to decline. The maximum geographic distance observed between the two Orobanche samples was 207 km. The black lines are whiskers indicating the magnitude of variation (Figure 5).

## 4. Discussion

### 4.1. SSR Transferability

One of the advantages of SSR markers is their higher level of polymorphism and reproducibility than RAPD and ISSR markers used to study the diversity of *O. crenata* in previous studies [12, 23–25]. The ability to transfer SSRs from one species to another depends on the primer sites flanking SSR motifs being conserved between the taxa [26]. Thus, the possibility of transferability of SSRs from closely related nonsource species becomes advantageous and minimizes the cost of SSR marker development. Closely-related species are more likely to share SSR primer sites than distantly related species; so, there could be a high level of polymorphic marker transferability exist [27].

Pineda et al. [27] conducted an SSR marker transferability test from *O. cumana* to *O. cernua* and reported high marker transferability with 92.4% (145) of the 157 SSRs tested as compared to the present study. The mean number of alleles per SSR locus (9.6) detected among the 96 *O. crenata* in the current study was higher than the mean number of alleles per locus (2.2) reported on *O. cumana* by Pineda-Martos et al. [15]. These differences of transferability and genetic variation can be explained by the closer phylogenetic relationship of *O. cumana* with *O. cernua* than *with O. crenata* and by the better resolution of capillary electrophoresis used in the present study than agarose electrophoresis used in Pineda-Martos et al. [15]. Similarly, Ludymila et al. *[*27*]* on *Coffea canephora* test 71 microsatellite markers originally developed for *C. arabica*, and 38 (53.52%) markers were successfully transferred for *C. canephora* and 20 primers were polymorphic.

In this study, the average of gene diversity was 0.82 with the maximum (0.93) at marker Ocum-059, and the minimum (0.64) was recorded at marker Ocum-063. The high level of gene diversity in this study was probably due to the presence of an extensive genetic diversity in the studied *O. crenata* genotypes that represented different geographic origins and linages.

The PIC value of SSR markers could vary between different species. The average PIC value of the SSR markers developed on *O. cumana* by Pineda-Martos et al. [15] was 0.52, which is less than (0.80) average PIC value obtained in this study. This difference may be due to different species and population size used in this study.

This study revealed high within population genetic diversity; among the four geographic populations, South Wollo accumulated more genetic diversity as compared to others. On the contrary, populations from North Wollo showed the least genetic diversity. Pairwise *F*_ST_ values between geographic origins of Ethiopian *O. crenata* populations also showed that low genetic differentiation among the population, which could be explained by the effect of low sample size on genetic diversity and exchange of contaminated seeds with neighboring regions, eventually leads gene flow among populations.

The principal component analysis reduced the variables into two important components. In the two-dimensional plot, subgroups contain different proportions of samples from different geographic sources and the presence of a random mixture of samples. Overall, principal component and STRUCTURE analysis result also confirmed that the members of each subgroup were collected from different areas of origin.

It is expected that *Orobanche* has a complex genetic structure because of the high level of outcrossing [29]. The structure analysis showed that when *K* = 2, the model-based clustering showed both subgroups consisted of samples from all regions without showing distinct correlation with the geographic population; on the contrary, a study conducted by Ennami et al. [30] showed a clear differentiation among Moroccan O. crenata samples according to the geographical origin. The absence of geographic-based grouping implies that there is a high intermixing of the gene pool of *O. crenata* in Ethiopia among populations. AMOVA also revealed high genetic diversity within populations than diversity among populations.

This higher variation within the population could be associated with gene flow, seed spread, and floral biology of the parasitic weed [8, 31]. In Ethiopian *O. crenata*, gene flow and the recent introduction of the parasitic weed reduce among population variations. Survey made by Besufekad et al. [32] indicated that the *O. crenata* appeared for the first time in 1983. Teklay et al. [10] explained that the distribution mechanisms of *O. crenata* in Ethiopia were through various ways like via contaminated seed exchange, free grazing, farm equipment, and wind. These could be cited as the major dispatch mechanisms that presumably resulted in gene flow among regions leading to less diversity among populations compared to the high within population variation. Besides, the absence of geographic-based genetic grouping could be explained due to an outcrossing nature of *O. crenata* species that leads to gene flow among the population that resulted in within postulation genetic diversity and lack of correlation between genetic and geographic distances for all the collected samples [24]. This is further supported by AMOVA and *F*-statistics result, which revealed a high percent value for within population diversity.

Other genetic diversity study on *O. crenata* populations from Israel, using RAPD marker [24]; from Egypt using ISSR Abdalla et al. [33] and RAPD [34]; from Spain and Israel using ISSR [12]; from Morocco using SRAP Ennami et al. [30], were also reported that there is high genetic diversity of *O. crenata* in their respective countries.

The statistical analysis computed to know how far the samples are isolated based on the geographic distance resulted in a significant correlation for samples collected in a range of 28 km. This result indicated that as the area of collection between two samples become wide apart, the genetic similarity between the samples started to drop in response to the geographic distance.

## 5. Conclusions

This study was able to identify 11 SSR markers that can be used for the future genetic diversity study of *O. crenata* populations. The markers used in this experiment were revealed significant genetic variation within O*. crenata* populations and able to structure the Ethiopian *O. crenata* genotypes into two genetic groups without following geographic origins. The low levels among population genetic diversity, indicating that resistance breeding for faba bean, can be done in one location. So far, in Ethiopia, the present study is the pioneer for *O. crenata* diversity analysis. To give a clear overview of population structure as well as the relationship among Ethiopian *O. crenata* populations, additional SSR markers should be either screened and/or characterized for further in-depth study of *O. crenata* to determine the effective population size and gene flow as an input to legume breeding activities, designing control, and management options.

## Figures and Tables

**Table 1:** List of transferred SSR markers used to test 96 *O. crenata* samples with major allele frequency, number of alleles, gene diversity, heterozygosity, and PIC values.

Locus	SSR sequence	Forward primer sequence (5′-3′)	Reverse primer sequence (5′-3′)	Average Tm (°C)	Expected size (bp)	Number of allele	Major allele frequency	Gene diversity	Heterozygosity	PIC value
Ocum-003	(GAT)_8_	CAAAGATGGTGGTTTTGCG	CTCGAACGCAAACTTTTGAA	55	94	7	0.4	0.77	0.72	0.75
Ocum-006	(CT)_8_	CTTATGTATGTTGTTCTTCTCTGCC	CATACATCCAATTAACATACAAGCA	57	90	7	0.34	0.8	0.63	0.77
Ocum-011	(CA)_8_	GCCGTGAACTCCACTACCAC	GAGTTAGGGTCAGTCTTGCGA	60	274	13	0.22	0.89	0.44	0.87
Ocum-023	(AG)_9_	CATCACCTCGAGTTTTCCGT	CGCAAGTTCACGAATTGAA	55	157	13	0.35	0.83	0.35	0.82
Ocum-043	(AGG)_10_	AGGTGCACTTAACCTTGACCTT	CTGCAGGTGGTCATGCTAGA	59	104	11	0.15	0.89	0.4	0.87
Ocum-052	(AG)_10_	CATGTCTAAGCTTTTGGCTCG	CAAGACTTGGAACAAGCAAATC	57	108	7	0.21	0.83	0.42	0.8
Ocum-059	(TC)_11_	TCTTGATTTGTATATGTCTGATGCAAT	ATGCTACAATAGAAATACACAACGAAC	56	90	17	0.13	0.93	0.43	0.92
Ocum-063	(AG)_11_	AACCAAGTTGATGCATCCGT	TCCCTCGGCATTCAGACTTA	57	90	4	0.53	0.64	0	0.59
Ocum-075	(CA)_12_	TGTGGATAGAGTATAAGCTACCAGTTC	TTCCCGTAGCTTGGAGAATG	60	110	10	0.22	0.85	0.32	0.82
Ocum-081	(CA)_13_	TTACAAGGTGAAACCACCCA	CAGCTACTGTCCGCAAGAAA	56	90	11	0.16	0.89	0.47	0.87
Ocum-094	(GT)_15_	TGGGAGCTTTGTACAGACACTG	GTTTTCTATTAAACCGTAACAAACTCT	58	141	6	0.33	0.77	0	0.73

**Table 2:** Genetic diversity estimates of regions of collection based on percentage polymorphism, allele numbers, observed and expected heterozygosity, allelic range, gene number and diversity, and Garza-Williamson index.

Region	*N*	%PL	*A* ^*n*^	Ho	He	*A* ^*r*^	*N* ^*g*^	*D* ^*g*^	*I*
South Tigray	30	45.45	8.82	0.39	0.82	24.73	60	0.79	0.46
South Wollo	31	72.73	8.54	0.37	0.82	22.37	62	0.82	0.49
North Wollo	5	63.63	3.45	0.34	0.69	15.54	10	0.74	0.41
South Gondar	30	54.54	8.18	0.39	0.80	24.09	60	0.75	0.45
Mean	24	59.09	7.25	0.37	0.78	21.68	48	0.77	0.45

Where *N*: population size, %PL: percent of polymorphic loci, *A*^*n*^: number of allele, Ho: Observed heterozygosity, He: expected heterozygosity, *A*^*r*^: Allelic range, *N*^*g*^: number of gene copies, *D*^*g*^: Average gene diversity over loci, *I*: Garza-Williamson index.

**Table 3:** AMOVA showing the distribution of genetic diversity within and among populations of Ethiopian *O. crenata* from different sources of origins.

Source	df	SS	MS	Variance	
				Estimated	%
Among population	3	37.423	12.474	0.120	2%
Among individuals within populations	92	654.442	7.113	2.559	55%
Within individuals	96	191.500	1.995	1.995	43%
Total	191	883.365		4.674	100%

Where df: degrees of freedom; SS: sum of squares and MS: mean squares.

**Table 4:** *F*-statistics values of populations collected from different region.

*F*-statistics	Value	P(rand ≥ data)
Fst	0.026	0.003
Fis	0.562	0.001
Fit	0.573	0.001

**Table 5:** Pairwise *F*_ST_ values between geographic origins of *O. crenata* populations.

	South Tigray	South Wollo	North Wollo	South Gondar
South Tigray	0.000	0.002	0.001	0.002
South Wollo	0.018	0.000	0.001	0.002
North Wollo	0.058	0.092	0.000	0.001
South Gondar	0.015	0.015	0.096	0.000

## Data Availability

The data used to support the findings of this study are available from the corresponding author upon request.
